# A Group-Based Telehealth Intervention for Birth Trauma: Protocol for a Pilot Feasibility and Waitlist Control Trial

**DOI:** 10.2196/69051

**Published:** 2025-09-23

**Authors:** Cassandra Sundaraja, Suzanne M Cosh, Amy Dianne Lykins, Hayley Farrell, Hira Masood, Melanie Kelly Williams, Jyoti Chaku, Joseph Turner, Anand Swamy, Phillip J Tully

**Affiliations:** 1 School of Psychology University of New England Armidale Australia; 2 New South Wales Department of Health Armidale Australia; 3 School of Rural Medicine University of New England Armidale Australia; 4 New South Wales Department of Health Newcastle Australia; 5 School of Psychology Deakin University Burwood Australia

**Keywords:** birth trauma, telehealth, group therapy, narrative therapy, early intervention, postpartum mental health

## Abstract

**Background:**

Traumatic childbirth experiences affect almost half of Australian women giving birth and can lead to significant mental health impacts, including postpartum depression, anxiety. and posttraumatic stress disorder (PTSD). Despite evidence supporting psychological interventions for birth trauma, there are prominent gaps in the accessibility of these treatments, particularly for postpartum women in regional or rural areas, who face long waitlists, geographical isolation, and high financial costs. Although narrative approaches hold promise for addressing birth-related trauma, no research study to date has specifically trialed a narrative-informed, group-based telehealth intervention in this space.

**Objective:**

This study aims to assess the acceptability and feasibility of a narrative-informed, group-based telehealth intervention for postpartum women in reducing the mental health impacts of having experienced a traumatic childbirth.

**Methods:**

This pilot feasibility trial with a waitlist control design evaluated a six-session narrative-informed, group-based intervention delivered weekly via telehealth to postpartum women who experienced a traumatic childbirth within the past 6 months. The intervention incorporated narrative therapy techniques, such as externalization, double-listening, and outsider witnessing. Participants from a specific catchment area of predominantly rural towns in New South Wales in Australia were randomly assigned to either an intervention group (IG) or a waitlist control group (WCG). Quantitative measures assessing mental health symptoms of postpartum depression (Edinburgh Postnatal Depression Scale [EPDS]), anxiety (Perinatal Anxiety Screening Scale [PASS]) and posttraumatic stress (City Birth Trauma Scale [City BiTS]) were administered prior to, in between, and at the end of treatment, and measures of client satisfaction (Client Satisfaction Questionnaire [CSQ-8]) and group cohesion (Group Cohesiveness Scale [GCS]) were administered on completion of the intervention.

**Results:**

The project was funded in March 2024. Recruitment was completed between July and August 2024. Eleven pretreatment sessions were held in August 2024. Of the 33 expressions of interest (EOIs) received by August 2024, 9 participants were recruited and randomized to the IG (n=4, 44.4%) and the WCG (n=5, 55.6%). The IG completed the six-session program between September and October 2024, with data collection finalized for pre-, mid-, and postintervention timepoints. The WCG began receiving the intervention mid-October 2024, with the final data collection in December 2024. Key feasibility and acceptability metrics include attendance rates, participant retention, and group cohesion scores. Data analysis is ongoing, with manuscript preparation planned for mid-late 2025.

**Conclusions:**

This study addresses a critical gap in evaluating scalable, accessible mental health interventions for birth trauma recovery. By using narrative approaches in a telehealth group format, this intervention directly responds to the barriers around accessibility and affordability highlighted in recent policy recommendations. Thus, findings from this pilot study could provide important directions in reducing the burden on perinatal mental health services in regional and rural Australia.

**Trial Registration:**

Australian New Zealand Clinical Trials Registry ANZCTR12624000460505p; https://www.anzctr.org.au/Trial/Registration/TrialReview.aspx?ACTRN=12624000460505p

**International Registered Report Identifier (IRRID):**

DERR1-10.2196/69051

## Introduction

### Background

Childbirth is a significant life event following on from pregnancy; however, almost half of the Australian birthing population (45.5%) reports having had a birth experience that they describe as traumatic and distressing [1]. Initially limited to physical injury during the birthing process, the current definition of a traumatic birth experience (interchangeably referred to as “birth trauma”) is “a woman’s experience of interactions and/or events directly related to childbirth that caused overwhelming distressing emotions and reactions, leading to short- and/or long-term negative impacts on a woman’s health and wellbeing” [2]. The risk of experiencing a traumatic childbirth increases when there is a history of previous trauma (including sexual trauma or birth trauma from a preceding birth experience); a history of mental health conditions; pre-existing disability; younger age; a lack of continuity of care (with respect to the availability of health professionals); having a lesbian, gay, bisexual, transgender, queer (or questioning), intersex, asexual, and other (LGBTQIA+) identity; living in a regional, rural, or remote area; and having a culturally or linguistically diverse background [3].

A wealth of literature links traumatic childbirth experiences or birth trauma to poorer mental health outcomes in postpartum women, including childbirth-related posttraumatic stress disorder (CB-PTSD), depression, and anxiety [4-9]. Further, the consequences of traumatic childbirth experiences have been shown to extend far beyond the immediate postpartum period and can also impact child outcomes [10]. Experiencing trauma symptoms postchildbirth has been linked to difficulties in breastfeeding [3,11] and, specifically, disruptions in mother-infant attachment and bonding [12-14]. These can have long-term adverse impacts on children’s socioemotional development [15,16] and long-term mental health [17], demonstrating the far-reaching impacts of traumatic childbirth experiences, if left unaddressed.

Given its widespread ramifications, the Parliament of New South Wales (NSW) formed a Select Committee in 2023 to conduct an inquiry into birth trauma. This is the first time in the world a parliamentary committee examined and reported on birth trauma. After reviewing over 4000 submissions from the community, the committee released a detailed report summarizing the submissions and hearings, as well as providing recommendations to reduce the likelihood of birth trauma and its adverse consequences [3]. Among various aspects pertaining to birth trauma, the report highlighted a dearth of adequate mental health services available for postpartum mothers, particularly in regional Australia, with long waitlists to see psychologists and expensive out-of-pocket costs for new parents [3]. This emphasized a critical need to offer accessible, cost-effective mental health services for postpartum mothers with traumatic childbirth experiences.

To address some of these barriers to accessing psychological interventions, such as long waitlists and higher costs, group-based interventions are a viable option for addressing mental health challenges in postpartum mothers, and have additional benefits of providing social support in the form of reducing feelings of isolation, having their feelings and experiences normalized and validated, and through modeling [18]. There has also been an increasing focus on telehealth interventions, necessitated by the COVID-19 pandemic. Research (including a systematic review and meta-analysis) has found that telehealth interventions are a feasible and effective option for supporting postpartum women with mental health diagnoses [19-21]. Taken together, group-based telehealth interventions have demonstrated increased attendance and comparable mental health outcomes to interventions delivered in person when treating mood and anxiety disorders in the general population [22,23] and have been successfully implemented to decrease symptoms of depression and posttraumatic stress in postpartum mothers as well [24]. This is significant, as psychological interventions that are group based and delivered via telehealth will greatly improve access to mental health services by overcoming established barriers to treatment, including affordability, availability, and convenience. For postpartum mothers in particular, such an intervention would further address additional barriers around time management, arranging for childcare, and needing to tend to their infants in an unfamiliar environment [25]. To date, no psychological intervention involving a telehealth group format for women with traumatic birth experiences has been studied.

Two systematic reviews [26,27] collated data from several studies that focused on the effectiveness of interventions to prevent CB-PTSD following birth trauma, ranging from primary prevention (antenatal education) to secondary prevention (hours to weeks following a traumatic childbirth) and tertiary prevention (when CB-PTSD is indicated). Most of the early intervention trials (ie, secondary prevention) focused on debriefing within 48-72 hours following birth or within 10 weeks postpartum, with no treatment effect on CB-PTSD [27]. The trauma-focused interventions reviewed included trauma-focused cognitive-behavior therapy (TF-CBT), individual or group based, predominantly with mothers whose infants were admitted to the neonatal intensive care unit or those with preterm infants, and eye movement desensitization reprocessing (EMDR) with women exhibiting symptoms of CB-PTSD [26,27]. A large proportion of these trauma-focused interventions demonstrated a positive treatment effect on CB-PTSD, with some of them reducing symptoms of postpartum depression or anxiety [26-28]. Specifically, for women at risk of or diagnosed with CB-PTSD, two Swedish studies trialed internet-based TF-CBT, with one study finding a positive treatment effect on CB-PTSD, depression, and anxiety [29] and the other finding no significant differences postintervention [30]. However, interventions such as TF-CBT and EMDR are required to be delivered by practitioners with specialized training in these modalities and therefore may be less available for postpartum mothers, particularly in rural areas. Further, these interventions have been predominantly trialed on women who are at risk of developing CB-PTSD or meet the diagnostic criteria for CB-PTSD versus women who report having experienced a traumatic childbirth, with or without symptoms of posttraumatic stress.

Women who report having had a traumatic birth experience frequently attribute their trauma to a lack or loss of control or sense of powerlessness [31], and birth storytelling has been found to be therapeutic for postpartum women, especially first-time mothers [32,33]. Therefore, a therapeutic approach incorporating narrative therapy techniques, such as externalizing the problems and reauthoring one’s story [34], which emphasizes self-empowerment, is likely to benefit this target population. Narrative therapy differs from other therapeutic modalities by explicitly acknowledging the social, cultural, and political contexts in which problems arise that ultimately shape the stories we tell ourselves [34]. As women’s childbirth experiences can be significantly impacted by sociocultural factors, political factors, and other practical concerns outside their control (eg, workforce shortages), a narrative-informed approach could be ideal in cultivating a strengths-based perspective on narratives. Narrative-informed interventions have been successfully used to address trauma delivered in group settings [35,36]. Linked to a narrative approach, a series of studies in Italy explored the use of a single instance of expressive writing within 48-96 hours postpartum, where women were asked to write about their labor and delivery experiences, focusing on their thoughts and feelings, the purpose of which was to develop a coherent narrative of their birth experience [37-39]. All studies indicated that expressive writing results in reduced psychological distress (depression and CB-PTSD) at 2-3 months postpartum [37-39].

Although curricula exist internationally for therapy groups for traumatic birth experiences [40], these have not been empirically evaluated. Therefore, this research will overcome this critical gap by assessing the acceptability and feasibility of a narrative-informed, group-based telehealth intervention for postpartum women in reducing the mental health impacts of having experienced a traumatic childbirth.

### Hypotheses/Research Question

Our first hypothesis is that a narrative-informed, group-based early intervention for postpartum women who have had a traumatic birth experience will be feasible and acceptable. Our second hypothesis is that the intervention will decrease symptoms of postpartum depression, postpartum anxiety, and/or postpartum post-traumatic stress disorder (PTSD) in women with traumatic birth experiences.

## Methods

### Study Design

This study is a pilot feasibility trial with a parallel design. Eligible participants will be randomly assigned to either an intervention group (IG) or a waitlist control group (WCG), with both arms delivered online and in group format. A WCG was included to ensure that all interested participants would have access to the intervention offered. This project was prospectively registered with the Australian New Zealand Clinical Trials Registry (ANZCTR12624000460505p) on March 26, 2024 (universal trial number U1111-1305-9340).

The pilot trial will be conducted entirely online using the videoconferencing software Zoom. Participants will be sent a password-protected URL to enter the videoconferencing room.

### Participants

The inclusion criteria were as follows:

- Women residing in a catchment area in Northern NSW, Australia, of predominantly rural towns spanning 131,785 km, with an estimated population of 962,390 residents as of 2021.
- Up to 6 months postpartum.
- Aged 18 years and above.
- Proficient comprehension of spoken and written English.
- Subjectively reporting having had a traumatic birth experience with their most recent birth.
- Having had a live birth with a gestation of at least 34+ completed weeks. (Births after 34 weeks of gestation are considered late preterm births, and infants may or may not require intensive care. Births prior to this gestation would likely require infants to be hospitalized and remain in intensive care and, therefore, would constitute a specific type of trauma. Such participants would likely benefit from a more homogenous group for increased group cohesion and support.)

The exclusion criteria were as follows:

- Women residing outside of the defined catchment area described earlier (Hunter New England Local Health District, NSW, Australia).
- Nulliparous, pregnant, or more than 6 months postpartum.
- Under 18 years of age.
- Not proficient in English.
- Do not subjectively report having had a traumatic birth experience with their most recent birth.
- Most recent delivery involving a still birth or a live birth with a gestation of under 34 weeks.
- Endorsing item 10 on the Edinburgh Postnatal Depression Scale (EPDS; pertaining to suicide risk) or have a total score of more than 13.
- Having serious mental health concerns that impinge on their ability to provide informed consent (eg, an intense mood or psychotic episode associated with bipolar disorder or schizophrenia).

### Sample Size

The study aimed to recruit 16 participants, which was deemed feasible within the project’s funding budget and timelines and is within the recommended size for group-based teletherapy [41]. As a pilot feasibility and acceptability study, should the study be deemed feasible and acceptable, the study findings may help inform estimates for sample size calculation for a larger trial. Consequently, no formal power calculation was performed for this study.

### Procedure

#### Recruitment

Recruitment took place in July and August 2024 and was completed by the start of September 2024. Participants were recruited through the following:

- Midwives and lactation consultants in public and private health care systems who aid postpartum mothers in recovery and breastfeeding.
- Child and family health nurses who provide free health and developmental checks on growth and development throughout infancy and early childhood. These nurses are also responsible for organizing local mothers’ groups for first-time mothers within a region.
- Perinatal Infant Mental Health Services (PIMHS), a free mental health service for women up to 2 years postpartum.
- Local referral hospitals (Armidale and Glenn Innes), who put the recruitment flyers in the personal health record books given to parents of all newborns born within that recruitment period.
- General practitioner obstetricians providing postpartum checks.
- Targeted advertising on social media (Facebook).
- Australian Facebook groups on birth trauma, breastfeeding, etc.
- Pamphlets with information about the study given to health care providers, including a QR code linking to an expression-of-interest survey.

### Ethical Considerations

This study will be conducted in compliance with all stipulations of this protocol, the conditions of the University of New England Human Research Ethics Committee approval (approval number HE24-054, valid till June 1, 2025), with site-specific approval granted to recruit from Hunter New England Health sites in the towns of Armidale and Glenn Innes; the National Health and Medical Research Council (NHMRC) National Statement on Ethical Conduct in Human Research; and the Australian Code for the Responsible Conduct of Research. The SPIRIT (Standard Protocol Items: Recommendations for Interventional Trials) 2013 checklist was followed when writing this protocol [42].

Potential participants who expressed interest were emailed a detailed information sheet by the research assistant and then invited to a pretreatment session. There was at least a week’s duration between receiving the information sheet and the pretreatment session for potential participants to consider participating. The pretreatment session included an introduction to the chief investigator (CI; author CS), who delivered the intervention and who presented screening questions to determine participant eligibility. The scope of the group therapy and guidelines for participation were explained (eg, telehealth etiquette, the need to be respectful, maintaining confidentiality of other group members, and distress management within sessions). At each individual pretreatment telehealth session, the information sheet was presented again, and participants got an opportunity to ask questions and clarify concerns, leading to the informed consent process. The clinician then gathered personal contact information and emergency contact details from the participants and explained the circumstances in which they would be used (ie, a participant leaves a group session abruptly, and one of the psychologists on the research team needs to do a telehealth welfare check to ensure safety). If deemed eligible, participants then signed the informed consent form, which could be done via an e-signature and sent as a file attachment within the password-protected Zoom Meeting or via email. The pretreatment session concluded with an assessment of the participants’ preferences for the session day and time. Additionally, participants also provided online implied consent before completing the pretest survey. Participants had the right to withdraw at any point without needing to provide a reason. Their identity was only known to the CI (CS) and the research assistant (author HM) to safeguard their privacy. All participants were remunerated with gift vouchers at designated intervals (to a total of AU $200, or ~US $130 at an exchange rate of AU $1=US $0.65, per participant) across the intervention to promote retention.

All data collected will be electronic and kept on the University of New England’s (UNE) centrally managed cloud server, as well as on a password-protected computer. Only the research team will have access to the data. All audio/video files recording the sessions will be deleted upon completion of the transcription process. Quantitative and qualitative data will be deidentified using numerical identifiers. The deidentified data will be stored in the UNE’s online research repository indefinitely and can be made available upon reasonable request.

### Allocation and Concealment

This pilot trial included an IG (who received the intervention immediately after recruitment) and a WCG (who were allocated to a waitlist during the same observation period as the intervention. Although there were no restrictions on outside care for the WCG, no intervention or support was actively offered to these participants. HM monitored the mental health of participants in the WCG (across the outcome measures administered at various timepoints; see Table 1) and alerted the team to any observed deterioration. A recent pilot feasibility trial conducted in Australia noted that people typically wait 6 months or longer to access mental health services [43]. Once the study with the IG concluded, the WCG received the full intervention, which involved a wait time of 6 weeks, significantly less than the typical waitlist.

A randomization sequence in alternating block sizes of 4 and 6 was generated from an online sequence generator by author PJT. The randomization sequence was revealed to the research assistant (HM) and only after completion of the baseline and eligibility measures. At that time, the research assistant informed participants of the intervention start date based on their allocation to either the IG or the WCG.

**Table 1:** Data collection timeline.

Measure	IG^a^ timepoints					WCG^b^ timepoints			
	T1^c^	T2^d^	T3^e^	T4^f^	T1		T2	T3	T4
City BiTS^g^	X^h^	X	X	—^i^	X		X	X	X
EPDS^j^	X	X	X	—	X		X	X	X
PASS^k^	X	X	X	—	X		X	X	X
GCS^l^	—	—	X	—	—		—	—	X
CSQ-8^m^	—	—	X	—	—		—	—	X

^a^IG: intervention group.

^b^WCG: waitlist control group.

^c^T1: prior to session 1 for the IG.

^d^T2: after session 3 for the IG.

^e^T3: after session 6 for the IG.

^f^T4: after session 6 for the WCG.

^g^City BiTS: City Birth Trauma Scale.

^h^X: applicable.

^i^Not applicable.

^j^EPDS: Edinburgh Postnatal Depression Scale.

^k^PASS: Perinatal Anxiety Screening Scale.

^l^GCS: Group Cohesiveness Scale.

^m^CSQ-8: Client Satisfaction Questionnaire.

#### Blinding

Participants were blinded to whether they were in the IG or the WCG. The CI was not involved in the randomization sequencing, nor in communicating intervention start dates to participants, depending on their group membership. However, as the CI was the clinician delivering the telehealth intervention, this was partial blinding. Participants filled in the measures at pre-, mid-, and postintervention timepoints using a unique identifier (generated by HM), so the survey data collected will be anonymous to the statistician.

Pre- (baseline), mid-, and postintervention measures were administered to the IG and WCG according to the schedule depicted in Table 1. The group-based intervention was conducted via telehealth to assess the feasibility and acceptability of a program that is likely to be cost-effective and accessible. The telehealth platform used in this study was a Zoom Conference Room, which was password-protected, so only individuals with the password (ie, study participants who met the eligibility criteria) were able to join in. For added security, all participants were sent two separate modes of communication, one with the Zoom Meeting link and another with the password to this meeting. Email reminders on the 2 days preceding each scheduled session were sent out to all participants. All sessions were recorded with informed consent. These were then transcribed verbatim for qualitative analyses to identify the key themes of healing from traumatic birth experiences.

### Intervention Details

The intervention was a group-based early intervention incorporating techniques and ideas from narrative therapy, roughly based on a modified version of the six-session trauma narrative recovery model developed by Lane and Lane [44]. The CI and one of the coauthors (HF) reviewed existing group-based and narrative-informed trauma-based interventions [40,44] and outlined each of the six sessions. A detailed treatment guide is freely available on an online repository for reproducibility of this intervention [45]. Each session ranged from 60 to 120 minutes; a large time range was deliberately proposed to enable participants to take breaks in the session, as needed, to attend to the needs of their babies (eg, a feed, nappy change). The intervention was conducted via a password-protected Zoom Conference Room. Table 2 provides an overview and brief description of each of the six sessions.

**Table 2:** Overview of the six sessions of the intervention.

Session number	Title	Description
1	Introduction and Psychoeducation	The first session introduces group members, recaps group guidelines, provides psychoeducation on birth trauma, and encourages reflection on personal experiences and coping strategies.
2	Sharing Birth Stories and Narrative Techniques	Participants commence sharing birth stories, with the clinician facilitating externalizing language use and implementing the “outsider witnesses” technique to enrich narratives.
3	Sharing Birth Stories and Narrative Techniques	Birth story sharing continues, focusing on externalizing problems and identifying unique outcomes in participants’ narratives.
4	Sharing Birth Stories and Narrative Techniques	This is the final session of birth story sharing, guiding participants to retell their stories with new perspectives and identified unique outcomes.
5	Reflection and Reauthoring	Participants reflect on shared experiences, rewrite or redraw their birth stories with new insights, and plan for the final session.
6	Integration and Closure	The group reflects on changes and emotional shifts, explores future directions, revises personal definitions, and completes a finishing ritual.

The designed intervention was carefully reviewed by an advisory group that consisted of a perinatal clinical psychologist, a narrative therapy clinician (experienced in working with women and trauma), a midwife, a doula (with lived experience from several years prior), and a person with recent lived experience. The group met twice (June and July 2024) and provided recommendations to modify the proposed intervention. Some of these recommendations were suggestions around the language used with participants (eg, changing the commonly used term “group rules” in group therapy to “group guidelines” to make it less authoritative and more collaborative), as well as activities participants could engage in during specific sessions (eg, ideas for “finishing rituals” to commemorate the end of the intervention). The advisory group met again on completion of the pilot trial to discuss findings, implications, and future directions.

#### Detailed Description of Sessions

##### Session 1: Introduction and Psychoeducation

The first group session (60-120 minutes) opened with introductions and ice-breaking activities to foster a comfortable atmosphere. The clinician presented the group guidelines to ensure a safe and supportive environment. Psychoeducation on trauma and birth trauma followed, with a specific focus on the postpartum period. The clinician then led a discussion on common trauma responses and their neurobiological basis, introducing the concept of healing from trauma through language and memory systems. Participants were encouraged to share coping strategies for triggers. The session included a reflection on personal definitions of “me” and “my space.” As an optional activity, participants engaged in a writing exercise to begin exploring their birth story.

##### Sessions 2-4: Sharing Birth Stories and Narrative Techniques

These sessions (60-120 minutes each) were dedicated to sharing birth stories, with 1 or 2 participants narrating their birthing experiences per session. Through careful questioning, the clinician helped participants use externalizing language to describe their problems (as separate from their personhood versus inherent in them). The clinician then implemented the “outsider witnesses” technique. This is a structured process wherein group members, acting as an audience to the story shared, are invited to reflect on and respond to a person’s story. This involves highlighting significant aspects that stand out, relating them to their own experiences, and describing how the story has impacted them, thereby potentially revealing alternative narratives or overlooked strengths. It usually involves a four-step process (identifying resonant expressions, recognizing values, making personal connections, and acknowledging impact) that validates the storyteller’s experience, while extracting moments of agency and resilience within their narrative. A key focus is the identification of unique outcomes in participants’ stories. The storyteller is then guided through a retelling of their story, incorporating new perspectives and the identified unique outcomes.

##### Session 5: Reflection and Reauthoring

The fifth session (60-120 minutes) began with completing any remaining story sharing, if needed. Participants were then guided to reflect on how their stories felt after sharing and witnessing others’ experiences. The clinician provided them with an opportunity to rewrite or redraw their birth stories, incorporating new insights. Next, the group collaboratively decided on the content for the final session and chose one or more finishing rituals to symbolize consolidation and the end of treatment.

##### Session 6: Integration and Closure

The final session (60-120 minutes) focused on instilling hope by reflecting on changes observed within oneself and others. The clinician guided a reflection on the evolution of birth stories and any emotional shifts experienced (if participants wrote down their birth story after session 1, this was used to facilitate this comparison). The discussion then turned to future directions, exploring values and sources of social support. Participants could revisit and potentially revise their personal definitions of “me” and “my space.” The chosen finishing ritual was completed, providing a sense of closure to the group experience. If desired, participants were given an opportunity to exchange contact information.

#### Outcomes

The following measures were administered according to the schedule depicted earlier in Table 1.

Treatment acceptability was assessed using the Group Cohesiveness Scale (GCS) [46] and the Client Satisfaction Questionnaire (CSQ-8) [47].

The GCS is a 7-item self-report measure assessing the level of cohesiveness in group therapy settings. The scale has strong psychometric properties and is a brief measure of group cohesiveness that can be used in psychiatric settings [46]. The CSQ-8 is a widely used 8-item scale measuring client satisfaction with mental health services. It is a subscale of the original Client Satisfaction Questionnaire-18, which has robust psychometric properties and, combined with its brevity, is a useful brief measure of client satisfaction [48]. The CSQ-8 has been used successfully in the provision of psychological interventions via telehealth (ie, videoconferencing) for conditions such as depression [49].

Treatment feasibility was assessed using indicators of treatment adherence and retention. Based on Wright et al’s [24] research on a virtual psychotherapy group for postpartum mothers, a rate of 70% was set to determine feasibility with respect to individual participant attendance across all the six sessions of the intervention, as well as overall retention (determined by the completion of all postintervention measures after session 6).

Postpartum depression was assessed using the EPDS [50], a 10-item self-report questionnaire widely used to screen for postpartum depression. It assesses symptoms experienced in the past 7 days, with higher scores indicative of a greater endorsement of depressive symptoms. The EPDS has demonstrated good reliability and validity in postpartum women [51] and has been validated for routine postnatal screening in Australia [52].

Postpartum anxiety was assessed using the Perinatal Anxiety Screening Scale (PASS) [53], a 31-item self-report questionnaire designed to screen for anxiety in both antenatal and postpartum women. Developed and validated for use in Australia [53], PASS provides cutoffs for differentiating between minimal, mid-moderate, and severe anxiety [54]. Its strong psychometric properties have led to its cross-cultural use and translation to other languages.

Posttraumatic stress symptoms were assessed using the City Birth Trauma Scale (City BiTS) [55], a 29-item self-report questionnaire specifically designed to measure postpartum PTSD following childbirth. It assesses symptoms based on the *Diagnostic and Statistical Manual of Mental Disorders, Fifth Edition* (DSM-5) diagnostic criteria for PTSD. The scale has shown good reliability and has been validated for use among Australian postpartum women [56].

### Data Analysis

#### Quantitative Data

All analyses will be based on the intention-to-treat principle. The quantitative data analyses will involve repeated-measures ANOVA entering the main effects for group, time, and group×time interaction, assuming α<.05 to denote a significant effect between the IG and the WCG for continuous study outcomes (City BiTS, EPDS, PASS). Assumption testing will examine normality using the Shapiro-Wilk test and sphericity using the Mauchley test of sphericity. Transformation and Huynh-Feldt correction will be applied, respectively, in the case of violation of these assumptions. The therapy process measures will be analyzed for between-group differences to determine the acceptability of the group intervention. As a feasibility and acceptability study, testing the proof of principle, our analyses will provide an estimate of mean (SD) change data and effect sizes to inform a larger trial in the future. The assessment of acceptability and feasibility postdelivery of the intervention, in both the IG and after the last session of the WCG, will afford the trial more quantitative data to identify aspects of the intervention that were perceived as beneficial and preferred.

#### Qualitative Data

Audio recordings of all group sessions were transcribed verbatim for qualitative analyses to identify the key themes salient in the treatment of traumatic birth experiences. The transcribed data from the six intervention sessions across both the IG and the WCG will be subjected to qualitative data analysis in order to better understand therapeutic processes that support change. Specifically, comprehensive process analysis (CPA) [57] will be adopted. CPA is an interpretive, qualitative research method used for analyzing therapy and is specifically designed to identify elements that could facilitate or hinder therapeutic progress [57]. The analysis will encompass three broad domains: context, key responses, and effects. Elliot et al [57] described four levels of *context* that must be explored: background (factors bringing participants into treatment, which in this study include experiences of birth trauma, previous coping styles, etc), presession context (referring to relevant events that could occur between sessions, eg, preparing a narrative of one’s birth story), session context (eg, therapeutic alliance and session tasks such as sharing one’s birth story, outsider witness), and, finally, episode context (conversational episodes pertaining to a significant event in therapy). An analysis of *key responses* relates to therapist or client responses perceived to be most helpful [57]. This analysis can include actions by the clinician or client, the style adopted, the nature of the content, and the quality of delivery. Finally, *effects* will be documented, aiming to track immediate and delayed consequences of the events within a session or across treatment.

Two coauthors will serve as CPA analysts (CS and HF), the former of whom is also the intervention facilitator. Both CS and HF are experienced in qualitative data analysis. Each analyst will independently review session transcripts (looking at the two initial sessions together, the two second sessions together, and so on) to identify significant process events and extract meanings with respect to context, key responses, and effects. The analysts will also maintain a reflective journal through this process. Once this step is complete, the analysists will meet in the presence of another coauthor (SC), also experienced in qualitative data analysis and not part of the intervention, who will assist in developing consensus through open dialogue, while ensuring that all perspectives are considered and discussed adequately. The multiauthor discussion and review, alongside considerations in the reflective journal, will support the trustworthiness and rigor of the analysis and ensure the analysis best reflects the data and process elements. With this process, we aim to produce a comprehensive understanding of which narrative intervention elements that effectively support birth trauma recovery in a telehealth group setting and which require modification, directly informing intervention refinement for the future. The embedded mixed methods design will allow details of the processes of change in the intervention to complement qualitative findings around feasibility and effectiveness. Outcome data will also be used to triangulate qualitative findings to further support which elements of change were and were not acceptable or useful for participants.

## Results

**Figure 1:**
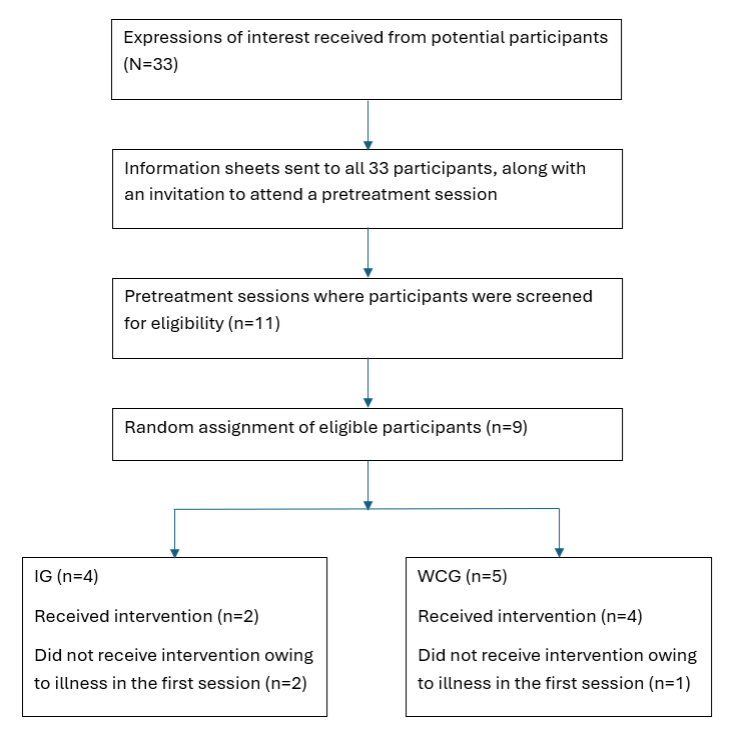
Flowchart of the pilot trial procedure. IG: intervention group; WCG: waitlist control group.

This project was funded in March 2024 through the Peregrine Centre Rural Mental Health Partnerships Small Research Grants (AU $26,707.68, or US $17,406.86) and was prospectively registered with the Australian New Zealand Clinical Trials Registry on March 26, 2024. Recruitment was completed between July and August 2024, and 33 expressions of interest (EOIs) were received, of which 12 (36.4%) booked in pretreatment sessions (n=17, 51.5%, did not respond to the invitation to join a pretreatment session, and n=4, 12.1%, replied but were ineligible to participate either due to age of the infant or location). In total, 11 pretreatment sessions were held in August 2024, and 9 (81.8%) participants returned signed informed consent forms (n=2, 18.2%, participants were deemed ineligible due to gestation at birth and age of the infant). These 9 participants (demographic details reported in Table 3) were randomly assigned to the IG or the WCG (see Figure 1). The IG completed the six-session program between September and October 2024, with data collection finalized for pre-, mid-, and postintervention timepoints. The WCG began receiving the intervention mid-October 2024, with the final data collection (timepoint 4) in December 2024. Data analysis is ongoing, with manuscript preparation planned for mid-late 2025. The study is proceeding according to protocol without significant deviations.

**Table 3:** Participant demographic information.

Participant	Group	Age (years)	Education level	Annual household income (AU $; US $)^a^	Parity	Age of infant (months)
P1	IG^b^	36	College certificate or diploma	160,000-169,999; 104,281-110,798	1	3
P2	IG	36	Undergraduate degree	60,000-69,999; 39,105-45,622	2	2
P3	WCG^c^	26	Year 12	80,000-89,999; 52,140-58,657	2	1
P4	WCG	Not disclosed	Not disclosed	Not disclosed	1	3
P5	IG	30	Postgraduate degree	170,000-179,999; 110,754-117,268	1	4
P6	WCG	20	Year 11	<10,000; 6515	1	2
P7	WCG	28	Undergraduate degree	130,000-139,999; 84,694-91,209	1	2
P8	IG	26	Year 10 or less	$100,000-$109,999; 65,152-71,014	1	<1
P9	WCG	Not disclosed	Not disclosed	Not disclosed	1	<1

^a^An exchange rate of AU $1=US $0.65 has been applied.

^b^IG: intervention group.

^d^WCG: waitlist control group.

To determine treatment acceptability, GCS-7 and CSQ-8 scores will be totaled for each participant, and a range of scores will be examined, including the central tendency score for each group. Higher scores will indicate greater treatment acceptability on both measures. Treatment feasibility will be determined by using attendance and retention metrics, with a threshold of 70% established as the criterion for feasibility drawn from recent research on virtual psychotherapy groups for postpartum mothers [24]. This threshold will apply to both individual participant attendance across all six sessions and overall retention, as measured by completion of postintervention measures across both groups. These predetermined benchmarks will provide clear criteria for determining whether the intervention warrants investigation in a larger trial.

The mental health outcome measures (EPDS, PASS, and City BiTS) will be scored according to the developer instructions. The last observation carried forward (LOCF) approach will be used to deal with any missing values, which involves replacing each missing value of a participant with the last observed value for that participant. The LOCF was chosen for its simplicity and frequent use in clinical trials [58]. As this is predominantly a feasibility and acceptability pilot study whose sample size is insufficiently powered to demonstrate statistically significant treatment effects, the LOCF was chosen over other more complex imputation methods that may not offer substantial benefits at this early stage. The average scores will be compared across the IG (who had these measures administered prior to, during, and after the intervention) and the WCG (who completed measures while they were on the waitlist, as well as after they received the intervention).

## Discussion

### Summary

This pilot trial will evaluate the acceptability and feasibility of a narrative-informed, group-based telehealth intervention specifically designed for postpartum mothers who have experienced birth trauma in the previous 6 months, and explore its potential impact on mental health symptoms of depression, anxiety, and posttraumatic stress. The findings from this study will have important implications for clinical practice, future research, and policy development in maternal mental health care.

Although research supports the use of psychological interventions in the postpartum period for mothers who are at risk of CB-PTSD, the majority of these studies tend to be individual based and in person [26,27]. Our intervention approach addresses the important barriers of accessibility and affordability by offering a group-based telehealth intervention that spans 6 weeks. Further, although narrative approaches, in the form of expressive writing, have been found to be useful in reducing psychological distress postpartum [37-39] and have been used in other trauma-focused group interventions [35,36], this is the first application of a narrative-informed group intervention for postpartum mothers specifically delivered through telehealth.

This pilot intervention also responds to some of the recommendations outlined following the NSW parliamentary inquiry, specifically pertaining to the need for more accessible and affordable psychological support services for people who experience traumatic childbirth experiences [3]. Currently, organizations in Australia that offer free postpartum mental health support report that their service capacity is restrained due to limited resources, with the main national helpline for postpartum mental health reporting that it has a capacity to attend to only 20%-30% of “live calls” on any given day [3]. Further, although the government does offer funding to access psychological services via Medicare rebates, there are several barriers and limitations; for instance, postpartum mothers cannot self-refer for these services (they require a referral from a general practitioner and other administrative procedures to be completed first), the funding is limited to 10 sessions per calendar year, and it is usually only sufficient to cover part of the costs [3]. Our telehealth delivery with a group format responds directly to address some of these accessibility barriers.

Apart from the cost of treatment, another frequently reported barrier among the perinatal population is the difficulty in attending mental health appointments due to varying reasons, including time constraints, childcare responsibilities, geographic distances (particularly in regional areas), transportation, and scarcity of qualified providers [59]. Providing an opportunity for postpartum women to access a telehealth psychological intervention mitigates several of these barriers, allowing them to access services from qualified professionals in a timely manner from the comfort of their own home.

As mentioned earlier, group-based interventions offer social support, normalization, and validation to participants [18]. However, this is not without disadvantages. Individual therapy can offer more privacy, individualized attention, and flexibility in scheduling (or rescheduling) sessions [18]. Mitigating some of these disadvantages should be the focus of future research.

### Dissemination and Scalability

Several avenues for dissemination have been planned, with some already implemented. The intervention was presented at numerous conferences, including those with practitioners and academic audiences both nationally and internationally. The treatment protocol was also presented to a group of psychologists in the public health sector in NSW, Australia, with further practitioner presentations planned into the future. A treatment guide has been developed in consultation with the advisory group, and this has been made available freely for practitioners to access [45]. Further avenues of dissemination will also be provided by the funding body. Finally, the authors will present a summary of the findings to participants who completed the intervention and will publish their findings in an academic journal.

The intervention was also designed to be scalable, and on reflection and in consultation with the advisory group, a practitioner treatment guide has been developed. For evaluating this intervention in larger samples (ie, multiple groups) in order to have statistical power, multiple clinicians will need to be familiarized with this treatment modality. The current intervention is designed such that anyone with training in mental health (ie, psychologist, psychotherapist, counsellor, narrative therapist, psychiatric social worker, or mental health nurse) will be able to implement it referencing the treatment guide, provided they have some experience and knowledge of group therapy, perinatal mental health, and narrative therapy techniques. Scaling up this intervention in a future study will require regular peer supervision for the clinicians involved, wider recruitment strategies, and robust screening and referral pathways for potential participants.

### Study Limitations and Future Directions

This study has several limitations that warrant consideration. First, the statistical power for detecting significant changes in the mental health outcome measures is limited owing to the small sample size (n=9) in this pilot trial, and the use of single imputation methods (eg, LOCF) to deal with missing values is generally not recommended as it is based on the assumption that outcomes do not change if a participant does not fill in a specific measure [58]. However, the primary aim of this project is to examine the feasibility and acceptability of delivering such an intervention, not to comment on significant treatment effects. Further, as we are adopting an embedded mixed methods approach, it is anticipated that the qualitative data will provide a rich understanding of the processes facilitating change as a result of the intervention. If feasibility and acceptability are demonstrated in this study, then future research can look at using this intervention in large-scale trials with a priori power calculations to evaluate statistical significance. In this instance, multiple imputation methods would be recommended to deal with missing values in order to provide more robust estimates.

Second, it is possible that there has been a selection bias owing to recruitment through social media and health care professionals. Potential participants were to send in an EOI if they wished to participate in the study and could have had higher digital literacy, less perceived stigma, and greater help-seeking tendencies than other eligible persons who did not submit an EOI. Further, postpartum women outside of the geographical region under study, and those with infants older than 6 months of age, were not eligible to participate in this study, which meant that the intervention could not be offered even when they reached out (sent an EOI). Participants who were deemed ineligible were provided with alternate mental health support resource options that they could access. This recruitment strategy also potentially limited the cultural diversity in potential participants. Future research with larger trials could seek to deliberately recruit participants from culturally diverse backgrounds, which could speak to this intervention’s broader applicability.

Third, the CI (CS) of this research project also delivered the intervention, given the resource constraints and need for a qualified professional to implement the intervention. To mitigate potential bias, CS was blinded to the random assignment of participants to the IG and the WCG; the research assistant (HM) handled all participant communication with respect to group allocation and the administration of outcome measures; and the coauthor (PT), who was not involved in the intervention delivery, will conduct the statistical data analysis.

Finally, our study does not involve long-term follow-ups beyond the intervention period, which limits our understanding of sustained effects of the intervention. This extended follow-up was not included in the study design, as the primary aims of the study were to assess feasibility, acceptability, and immediate impacts on mental health measures. However, future research with larger samples would benefit from including follow-ups a few weeks or months after the intervention to determine whether any changes in symptoms were sustained.

### Conclusion

This pilot feasibility trial aims to address significant barriers in mental health support services available for women who experience a traumatic childbirth experience by evaluating a narrative-informed, group-based telehealth intervention delivered within 6 months postpartum. Although this study is preliminary and limited by some aspects of methodology (including a small sample size, partial blinding, and the lack of follow-up), it still establishes the groundwork for developing a scalable, evidence-based intervention that can be integrated into existing perinatal mental health pathways to mitigate the impacts of traumatic childbirth experiences.

Our research directly responds to some of the recommendations from the NSW Parliamentary Inquiry into Birth Trauma [3] specifically addressing the urgent need for accessible and affordable psychological support for postpartum mothers, particularly in regional and rural areas. The uniqueness of its contribution lies in its application of narrative therapy principles within a telehealth group format, specifically using technology to overcome geographical, logistic, and financial barriers that postpartum women in regional and rural Australia face. It is also an intervention that various mental health clinicians could relatively easily learn to implement (versus other trauma-focused interventions that require delivery by psychologists with additional training), which in turn could contribute to reducing the disease burden and the drain on the health system, currently plagued by long waitlists and a limited number of clinicians able to offer mental health services. Finally, if feasibility and acceptability are demonstrated, future research can look at larger, adequately powered randomized controlled trials to evaluate efficacy, including long-term follow-ups and exploring implementation in diverse populations.

## Abbreviations

CB-PTSD: childbirth-related posttraumatic stress disorder
CI: chief investigator
City BiTS: City Birth Trauma Scale
CPA: comprehensive process analysis
CSQ-8: Client Satisfaction Questionnaire
EOI: expression of interest
EMDR: eye movement desensitization reprocessing
EPDS: Edinburgh Postnatal Depression Scale
GCS: Group Cohesiveness Scale
IG: intervention group
LOCF: last observation carried forward
NSW: New South Wales
PASS: Perinatal Anxiety Screening Scale
PTSD: posttraumatic stress disorder
TF-CBT: trauma-focused cognitive-behavior therapy
WCG: waitlist control group

## Data Availability

Deidentified data and materials will be placed in an online research repository, which will be made available upon reasonable request on completion of the pilot trial.
